# Comparative phenotypic and transcriptomic analysis of Victoria and flame seedless grape cultivars during berry ripening

**DOI:** 10.1002/2211-5463.12996

**Published:** 2020-11-04

**Authors:** Haixia Zhong, Fuchun Zhang, Mingqi Pan, Xinyu Wu, Wen Zhang, Shouan Han, Hui Xie, Xiaoming Zhou, Min Wang, Caikasimu·maikeer Ai, Tianming He

**Affiliations:** ^1^ College of Forestry and Horticulture Xinjiang Agricultural University Urumqi China; ^2^ Institute of Horticulture Crops Xinjiang Academy of Agricultural Sciences Urumqi China

**Keywords:** berry ripening, gene expression, grapes, sugar accumulation, transcriptome sequencing

## Abstract

Grape berry development is a highly coordinated and intricate process. Herein, we analyzed the phenotypic and transcriptomic patterns of Victoria (VT) and Flame Seedless (FS) grape varieties during berry development. Physiological analysis and transcriptomic sequencing were performed at four berry developmental phases. VT berry size was comparatively larger to the FS variety. At maturity, 80 days postanthesis (DPA), the FS soluble solids were 61.8% higher than VT. Further, 4889 and 2802 differentially expressed genes were identified from VT and FS 40 DPA to 80 DPA development stages, respectively. VvSWEET15, VvHXK, and MYB44 genes were up‐regulated during the postanthesis period, while bHLH14, linked to glucose metabolism, was gradually down‐regulated during berry development. These genes may have significant roles in berry development, ripening, and sugar accumulation.

## Graphical abstract

Grape berry development is a highly coordinated process. Herein, phenotypic and transcriptomic patterns of Victoria and Flame Seedless varieties were analyzed during berry development. Physiologically, VT berry size was larger than the FS variety, but FS contained higher levels of soluble solids at maturity. Furthermore, we examine genes that have significance in berry development, ripening, and sugar accumulation for the two varieties.

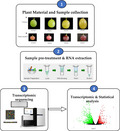

**Abbreviations**
cDNA: complementary DNA
DEG’s: differentially expressed genes
DNA: deoxyribonucleic acid
DPA: days postanthesis
FPKM: fragments per kilobase of transcript per million
FS: flame seedless grape
qRT‐PCR: quantitative reverse transcription polymerase chain reaction
RNA: ribonucleic acid
VT: victoria grape

Grape (*Vitis vinifera* L.) is an important horticultural crop in tropical and sub‐tropical regions. It is an economically significant fruit species worldwide, especially in the Mediterranean region, Asia, and central Europe where it is grown [1]. The fruits are extensively utilized for fresh use, raisins, juices, wines, et cetera.

Typically, grape berries growth as well as development are very dynamic and it is classified into three developmental phases [2]. The initial fruit developmental phase involves a notable increase in berry size, up to nearly 70% of its total fruit volume. Thereafter, the fruit enters a lag phase developmental stage where the berry size nearly stagnates but its seed embryo and coat develops, allowing accumulations of acids, aroma, and flavor compounds [3]. Toward the end of this phase, the berry enters the veraison stage. It is here where fruits begin to accumulate its coloration among the colored grapes and subsequently enter the second developmental and ripening phase. At this point, the berry restarts broadening, flavor and phenolic compounds, sugars and aroma start to build up, while organic acids and chlorophyll contents significantly decline. Hydrolase activity rises as the cell wall is altered, presenting a decrease in pectin and initiating fruit softening. At the end‐point maturity stage, the berries are colored in red‐skinned varieties, juicy, soft, and edible [4, 5].

In fruit productivity, sugar accumulation is considered as a very important aspect that defines high‐quality traits. Thus, it is used as a vital index in evaluating the quality of grape berries [6, 7]. Therefore, research on the regulation mechanisms of fruit quality, especially on sugar accumulation and regulation mechanisms between different fruit varieties, is of great significance to the improvement of grapefruit quality and the formation of regional adaptive and favorable characteristics that promote efficient, healthy development, and growth of the grape industry [7]. In grapes, the main soluble sugars are glucose, fructose, and sucrose, which undergo alteration during the berry developmental stages as a result of change in metabolism‐related enzymatic activities. For example, the activity of sucrose synthase (SS) and acid invertase (AI) determines the strength of the organ bank of grape berry and is higher in the early development stage of grape berry, but lower in the mature stage [8]. Neutral invertase (NI) and AI play significant roles in the sugar accumulation process, with respective negative and positive correlation in sucrose accumulation, together with fructose and glucose accumulation [9]. In fruit ripening process, the AI activity is always at a higher state, while the SS and sucrose phosphate synthase (SPS) activities always undergo gradual increased with the accumulation of sugar in fruits. Hexokinases (HXK) involved in the sugar signal transduction process and fructokinases (FRK) which catalyze the basic irreversible phosphorylation of fructose and glucose also have been identified [10]. Therefore, different kinds of fruits have different metabolic enzyme activities, with a certain relationship between them, which acts on the accumulation of fruit sugar during its development.

The release of the grape reference genome [11] and the conception of new transcriptomic tools have facilitated an in‐depth genome‐wide advancement study of gene expression, dynamic, in grape berries, and other fruits [6, 23]. A study by Lin *et al* on fruit maturation of *Citrus reticulata* showed that the inclusion of SPS gene and expression of specific enzymes in the up‐regulated varieties increased during fruit maturation, thus indicating that certain enzymatic activities influence accumulation and degradation of sugar and organic acids, respectively. In grape berry, starch granules were shown to be located in the subepidermal tissues at the chloroplasts, acting as the temporary reserves for physiological processes such as photosynthesis, and during that, different enzymes activities and gene expression were elevated in starch synthesis. This focus on the morphologies, enzymological, and transcriptional analysis provided knowledge on the key role of starch during maturation, and ripening of berries and their quality [24]. The use of metabolomics and micrometeorology has also offered insights on accumulation of metabolites, both primary and secondary, of grape‐cluster pattern as a function of spatial variations, which improves knowledge on the modulation of berry metabolism in warm and arid/semi‐arid areas by regulating sunlight so as to accurately modulate fruit composition [25]. Further study efforts have also been directed toward understanding the grape vine berry quality, softening, and cracking, as well as skin coloration [26, 27]. The use of transcriptome and functional analysis during grape berry ripening has revealed numerous enzymes during fruit development and suggested an increased gene activities associated with respiration and metabolic pathways throughout the ripening stage [28]. Furthermore, specific ATPases and malate transporters have been shown to display different development and temperature‐dependent expression patterns, and adding to the direct effects by different sizes are key aspects involved in regulating ripening and are likely to contribute to the varying qualities of the berries [23, 29].

Past studies on grapes’ berry development involved single varieties from over a wide geographical range [13, 18, 19, 23, 27]; hence, it is difficult to evaluate the genotype‐specific effects. Herein, we applied two diverse grape varieties cultivated in the same region and conducted an in‐depth comparative transcriptomic analysis at four fruit developmental stages, concentrating on traits related to the sugar accumulation in grape berries. The grape varieties used are Victoria and Flame Seedless.

## Materials and methods

### Plant material and sample collection

**Fig. 1:**
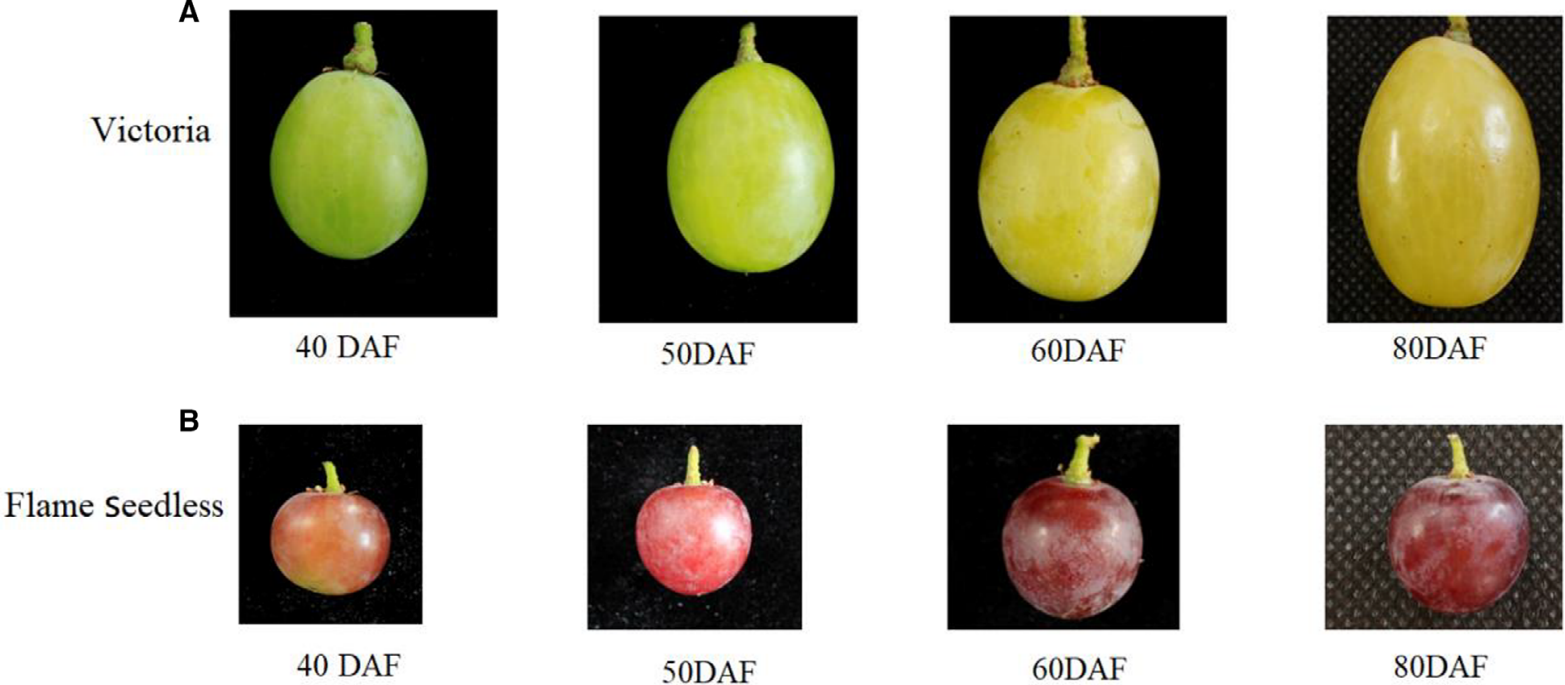
Distinct morphological characteristics of grape berries at 4 different developmental stages. (A) Victoria (VT) cultivar; (B) Flame Seedless (FS) cultivar.

The *Vitis vinifera* L. cultivars, Victoria (hereafter referred as VT) and Flame Seedless (hereafter referred as FS) Plant materials, with stable soluble solid contents of 6 years old vineyards, were obtained from grape base of Anning Canal Experimental Farm at the Horticulture Institute of Xinjiang Academy of Agricultural Sciences, coordinates longitude 87°28′10″ East, latitude 43°35′10″ North. The test field is located in a semi‐arid region and is made up of sandy loam soil with a pH 8.0. The plants were cultivated at row spacing of 1 × 3.5 m, with the vine form being on a single cordon along the ditch. Soil fertilizer application and water management practices were routinely carried out as per set in‐house procedures. The transcriptome of grape cultivars VT and FS was evaluated during fruits development, with reference to characteristic physiological changes. The first developmental stage, at 40 days postanthesis (DPA); the second stage at 50 days postanthesis (DPA); the third stage at 60 days postanthesis (DPA); and the fourth stage at 80 days postanthesis (DPA) for both the VT and FS grape varieties (Fig. 1A,B). During planting stage, 15 plants with almost the same growth status as our experiment materials were selected. Thereafter, three plants from these 15 plants were randomly selected. In each replicate, a pool of 60 berries was collected from the up, middle, and lower part of the same cluster in one plant. In triplicates, the fruit samples were collected at four stages: 40DPA, 50DPA, 60DPA, and 80DPA.

### Determination of the content of soluble solids

The content of Soluble Solids was measured at different developmental and ripening phases of fresh grapefruit samples. Japanese PAL‐1 Digital visible soluble solids meter was used, and the results were expressed in ºBrix.

### Total RNA extraction, purification, cDNA library preparation, and sequencing

Spin Column Plant total RNA Purification Kit was used to extract total RNA, as per the manufacturer’s protocol (Sangon Biotech, Shanghai, China). The purity assessment of the extracted RNAs was performed on a 1% agarose gels and then followed by NanoPhotometer spectrophotometer (IMPLEN, Los Angeles, CA, USA). The quantification of RNAS was performed using Qubit RNA Assay Kit in Qubit 20 Fluorometer (Life Technologies, Carlsbad, CA, USA). The RNA integrity was subsequently assessed by the RNA Nano 6000 Assay Kit of the Agilent Bioanalyzer 2100 system (Agilent Technologies, Santa Clara, CA, USA). Next, Sequencing libraries were created using NEB Next Ultra RNA Library Prep Kit as per the manufacturer's instructions where code indexes were added to each sample. The mRNA was briefly purified from 3 μg total RNA from each of the three replicates using poly‐T oligo‐attached magnetic beads and then broken down to short fragments used to synthesize first‐strand cDNA. The enzymes, DNA Polymerase I and RNase H, were used to synthesize the second strand of cDNA. Polymerase chain reaction was carried out with Phusion High Fidelity DNA polymerase where universal PCR primers and index (x) primer were used. Finally, six paired‐end cDNA libraries with an insert size of 300 bp were constructed for transcriptome sequencing and sequenced on Illumina HiSeq X‐ten platform (alumina Inc., San Diego, CA, USA) by Biomarker Technology Corporation (www.biomarker.com.cn).

### Bioinformatics analysis

#### Quality control

Through in‐house developed Perl scripts, raw data (raw reads) of fastq format were processed first. In this step, clean reads (clean data) were obtained by removing reads containing poly‐N, adapters, and low‐quality reads from the raw data. At the same time, phred scores of Q20 and Q30, GC content, and sequence duplication level of the clean reads were calculated and the downstream analyses were based on clean reads with the highest quality.

#### Differential gene expression analysis

The clean reads obtained was assembled using sting tie V1.3.1 [30], and thereafter, the sequence reads mapped to the *Vitis vinifera* reference genome (PN40024 12X.v1) [11], using HISAT2 [31], with default settings. FPKM evaluated gene expression levels in each sample. To measure the FPKM value and screen out the DEGs, edge r software [32] was utilized. Genes with FPKM < 0.1 in every sample dataset were excluded prior to this analysis. Differentially expressed genes were determined by analyzing the results based on the foldchange (FC ≥ 2 or ≤ 0.5) and false discovery rate (FDR < 0.05). Gene ontology (GO) analyses were used to calculate the functional category distribution frequency and to predict the gene function using DAVID bioinformatics resources [33]. The Pearson correlation coefficient (PCC) calculations of the DEGs were used to construct networks where Venn diagrams were built using the online available tools, Venny (http://bioinfogp.cnb.csic.es/tools/venny/).

### qRT‐PCR validation

Primer‐BLAST online software was used to design gene‐specific primers for qRT‐PCR analyses (Table S1). These genes were randomly selected, and *GADPH* was utilized as the housekeeping gene in the validation step.

Grape pulps were ground to a fine powder in liquid nitrogen. Thereafter, RNA was extracted using the TRIzol Plus RNA Purification Kit (Invitrogen, San Diego, CA, USA) as per the manufacturer’s guidelines. The RNA was stored at – 80 °C until further use.

The first‐strand cDNA was synthesized using M‐MLV reverse transcriptase kit (TaKaRa, Dalian) according to the manufacturer’s instructions. PCR assay was performed under the following conditions: 95 °C for 2 min followed by 40 cycles at 95 °C for 5 s, 60 °C for 30 s, 72 °C for 10 s, and thereafter 72 °C for 10 min; RT‐qPCR QuantiNova SYBR Green PCR Kit (Qiagen, Hilden, Germany) and LightCycler96 (Roche, USA) were used in this step. The relative expression of mRNA was calculated using the 2^−∆∆C^T method, and graphpad prism 8.0 software (GraphPad Software, Inc., San Diego, CA, USA ) was utilized for statistical analysis. The experiments were conducted in triplicates.

### Statistical analysis

All values obtained were presented as mean ± SD. The significance of differences between means was determined by Student’s *t‐*test for comparison purposes. Values with *P* < 0.05 were regarded as statistically significant.

## Results

### Phenotypic features of VT and FS GrapeFruits

The transcriptome of grape cultivar VT and FS was evaluated during fruits development, with reference to characteristic physiological changes. The first developmental stage being at 40 DPA, followed by the second stage at 50 DPA, third stage at 60 DPA, and the final fourth stage at 80 DPA for both the VT and FS grape varieties (Fig. 1A,B). The berry size and berry shape index characteristics of VT and FS were assessed and are described in Table 1.

**Table 1:** Berry size and berry shape index characteristics of two varieties at maturity (80DPA)

Variety	Length (mm)	Diameter (mm)	Shape Index	Average grain weight (g)
Victoria	33.02	23.03	1.43	10.90
Flame seedless	17.13	16.95	1.01	3.32

### Variation and comparison of soluble solids content during berry development of two grape cultivars

**Fig. 2:**
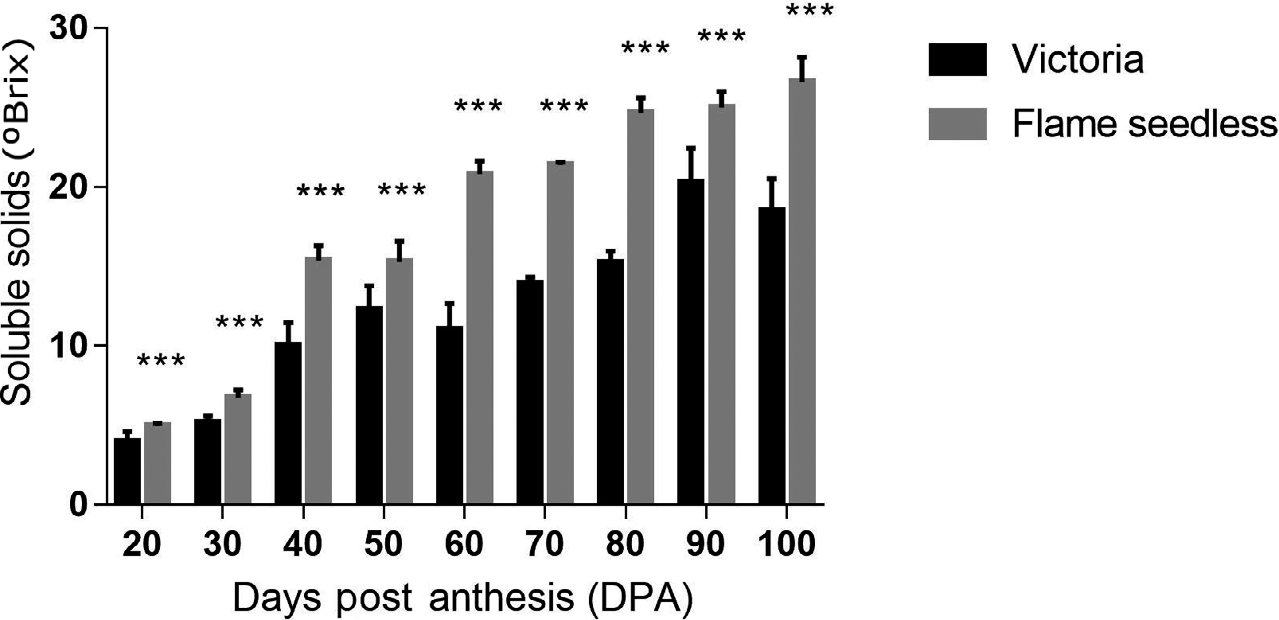
Variation and comparison of soluble solids content in the development of VT and FS grapefruit. Two‐tailed *t*‐test was used, with 5 biologically independent replicates. Error bars represent SD. *P*‐value < 0.0001.

The VT and FS are both early ripening varieties. From the study findings, it was evident that the content of soluble solids in each fruit variety gradually increased during their fruit development phase. There was a significant difference in soluble solid content between VT and FS fruits during development and maturation. During the same period, the soluble solids content of FS grapefruit was significantly higher, compared to Victoria. At point of fruit maturity, 80DPA, the FS soluble solids were 61.8% higher than VT (Fig. 2).

### Sequencing summary of all the libraries

To further illuminate the molecular basis for regulation difference between VT and FS, we performed a comparative transcriptome analysis through RNA sequencing. A total of 24 cDNA libraries were constructed using the berries from VT and FS varieties 40, 50, 60, and 80 days postanthesis, with each test being done in three biological replicates.

A total of 428.37G raw data were obtained from the generated libraries. The average sequencing error rate was 0.029% while the average GC content for each sample was above 45.88% (Table S2). The data ratio of sequencing quality to Q30 base was above 88.74%. The obtained transcripts were mapped to the *Vitis vinifera*. The alignment efficiency between reference genome and reads of each sample ranged from 57.88% and 83.94% (Table S3). Genes with normalized expression values of FPKM < 0.1 were considered as ‘too low expressed’ and were excluded in downstream analysis.

**Fig. 3:**
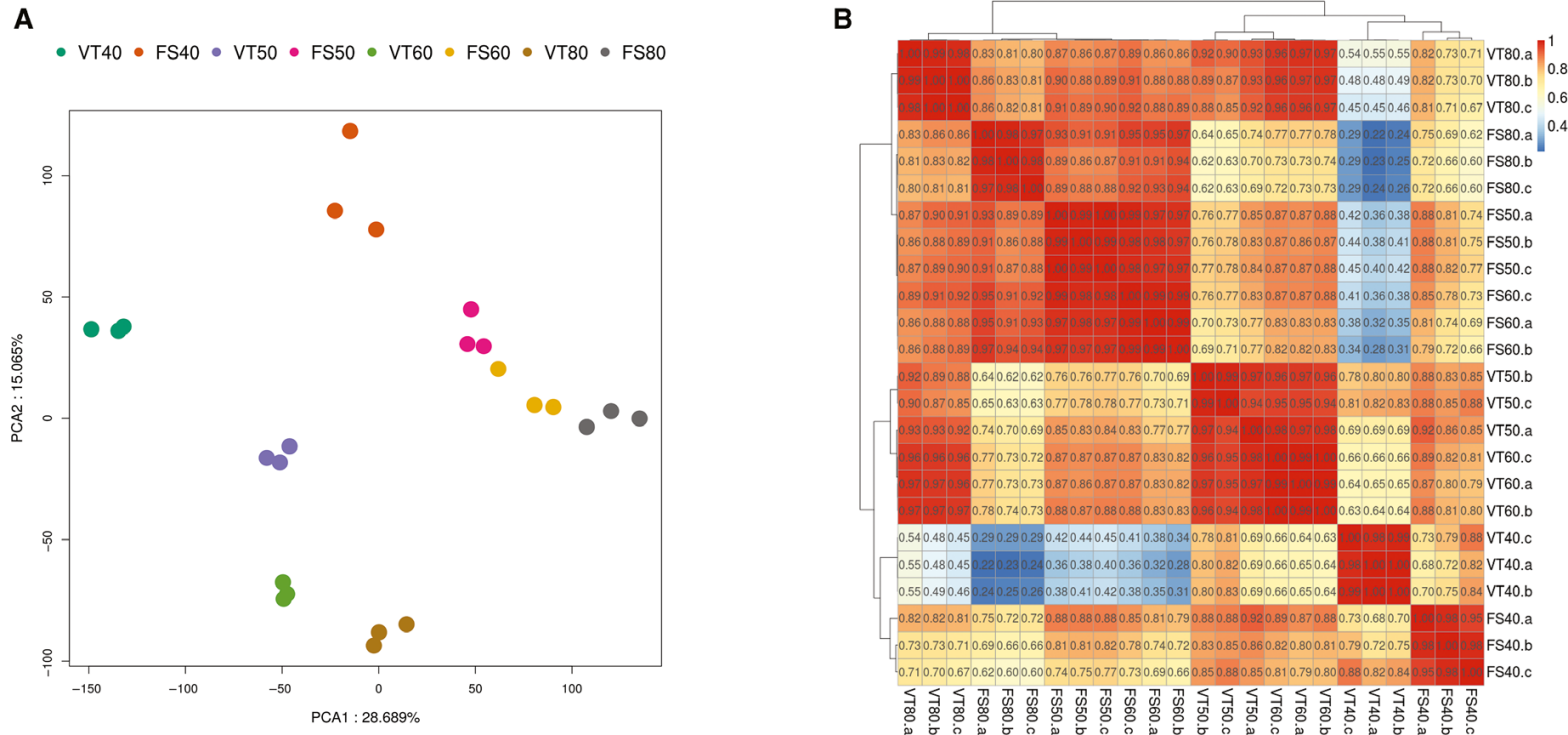
The discrete expression patterns of mRNAs. (A)Principal components 1 (PC1) and 2 (PC2) of the expression values for all genes in Victoria (VT) cultivar and Flame Seedless (FS). (B) Heatmap showing the sample correlation analysis of all the 24 sequenced samples.

In order to understand the spatiotemporal expression patterns of all samples, principal component analysis (PCA) was performed. The three samples in the same timepoint could form independent clusters (Fig. 3A,B).

### DEG’s between VT and FS groups

**Fig. 4:**
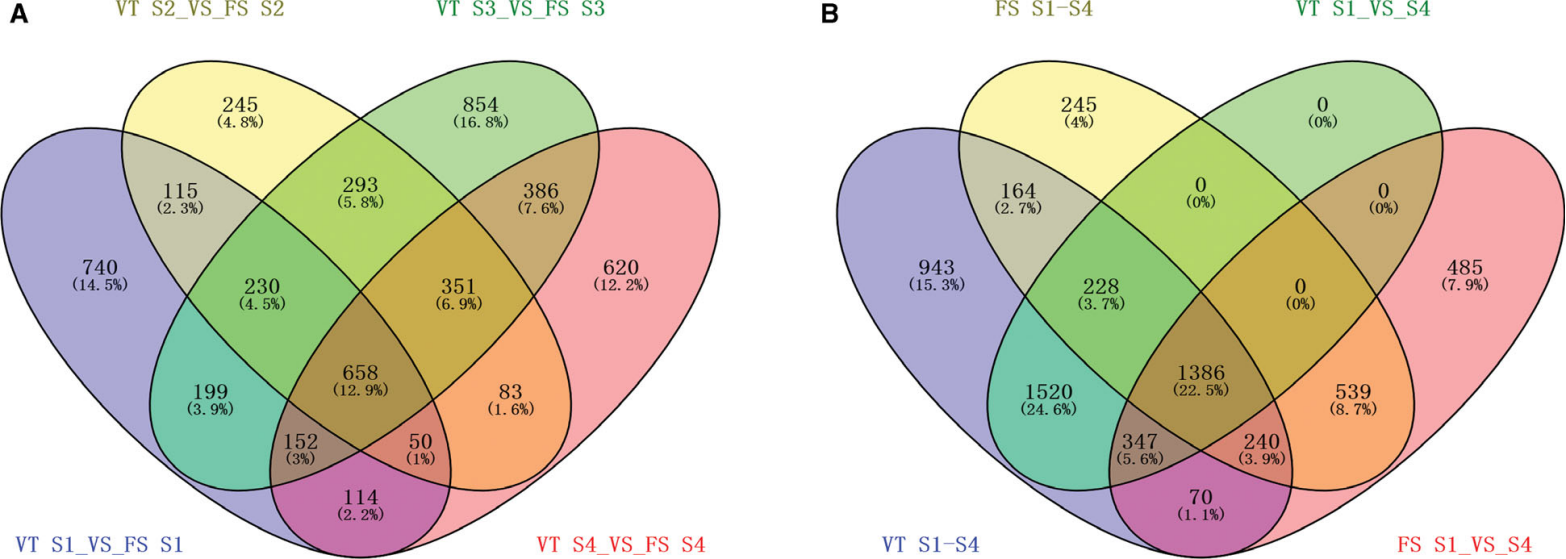
Venn diagram illustrating the differentially expressed genes of Victoria (VT) and Flame Seedless (FS) grape fruits. (A) Comparison of differentially expressed genes between VT and FS varieties at the same period (S1 VT/FS,S2 VT/FS,S3 VT/FS,S4 VT/FS); (B) Comparison of differentially expressed genes between the two cultivars from the first to the fourth fruit development stages.

Differentially expressed genes (DEG) in the different developmental stages of VT and FS were analyzed, and a Venn diagram was used to illustrate the unique or commonly expressed gene features (Fig. 4A,B). In total, 9820 DEG’s were identified from the grapefruits of VT and FS. Specifically, in the four fruit developmental stages (40 DPA, 50 DPA, 60 DPA, and 80 DPA), there were 658 common differential genes shared by the two varieties. At 40 DPA, VT and FS had 2258 differential genes, of which 970 were up‐regulated, 1288 were down‐regulated, and 740 were differentially expressed. At 50 DPA, VT and FS had 2025 differential genes, of which 714 were up‐regulated, 1311 were down‐regulated, and 245 were differentially expressed. At 60 DPA, VT and FS had 3123 differential genes, including 1146 up‐regulated genes, 1977 down‐regulated genes, and 854 DEG’s while in the 80DPA, VT and FS had 2414 differential genes, of which up‐regulated genes were 953, 1461 genes were down‐regulated, and 620 differential genes were differentially expressed. VT and FS had a gene difference of 1053 in 40DPA and 50DPA developmental timepoints, whereas there was a common gene difference of 1532 for the 50DPA and 60DPA timepoints, and in 60DPA and 80DPA timepoints. Out of 1547 differential genes, 1239 differential genes were shared between 40DPA and 60DPA, 974 differential genes shared between 40DPA and 80DPA, and 1142 differential genes shared between the 50DPA and 80DPA fruit developmental stages (Fig. 4A).

A total of 4889 differential genes were identified in the VT fruit development from 40 DPA to 80 DPA, whereas a total of 2802 differential genes were found in the FS fruit development from 40 DPA to 80 DPA. The two grapefruit varieties shared 1386 differential genes between 40 DPA to 80 DPA fruit development stages. In VT 40DPA compared with 80DPA, there were 3841 differential genes, of which 245 were differentially expressed. In the FS 40DPA, compared with 80DPA, there were 3067 differential genes of which 485 were differentially expressed. There were 943 differential genes specific in VT 40DPA and 80DPA, compared to other groups (Fig. 4B).

### Gene function annotation and enrichment analysis of DEG’s

Gene function annotation of differentially expressed genes was attained by searching the obtained transcripts against the COG, GO, KEGG, NR, KOG, Pfam, Swiss‐Prot, and EggNOG databases. In sum, 29 131 unigenes were successfully matched with at least one annotation database (Table 2).

**Table 2:** Statistical table of the number of differentially expressed genes annotated

DEG Set	Total	COG	GO	KEGG	KOG	NR	Pfam	Swiss‐Prot	EggNOG
VT_vs_FS_40DPA	2214	952	1932	767	1144	2214	1816	1651	2103
VT_vs_FS_50DPA	1998	855	1770	644	997	1998	1643	1502	1900
VT_vs_FS_60DPA	3075	1296	2674	1032	1585	3075	2511	2253	2943
VT_vs_FS_80DPA	2388	1034	2101	853	1217	2388	1953	1756	2273
40DPA_VS_50DPA_VT	1770	732	1559	607	860	1770	1478	1345	1698
40DPA_VS_60DPA_VT	2500	972	2181	834	1257	2500	2077	1880	2397
40DPA_VS_80DPA_VT	3430	1456	3022	1150	1761	3430	2874	2604	3295
50DPA_VS_60DPA_VT	744	280	636	229	352	744	620	566	711
50DPA_VS_80DPA_VT	1667	680	1486	528	819	1667	1434	1302	1600
60DPA_VS_80DPA_VT	1399	569	1226	416	647	1399	1183	1085	1342
40DPA_VS_50DPA_FS	928	388	834	327	467	928	806	743	904
40DPA_VS_60DPA_FS	1823	749	1604	613	900	1823	1547	1391	1760
40DPA_VS_80DPA_FS	3022	1289	2665	1031	1531	3022	2561	2290	2912
50DPA_VS_60DPA_FS	444	174	371	148	203	444	376	333	425
50DPA_VS_80DPA_FS	1261	502	1100	424	591	1261	1077	967	1208
60DPA_VS_80DPA_FS	468	192	400	139	200	468	408	347	449

**Fig. 5:**
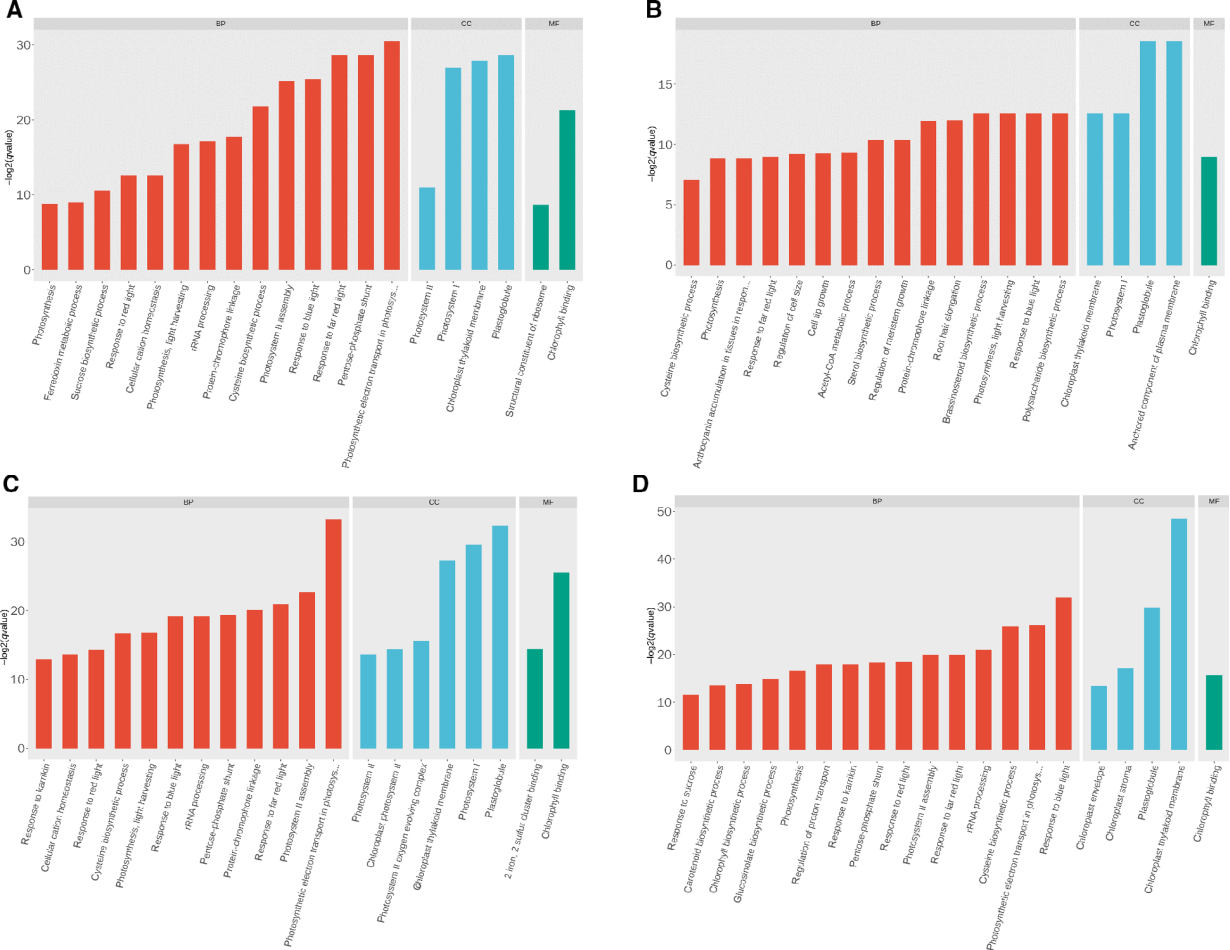
GO enrichment results for each group of differential genes (A: VT40DPA versus FS 40DPA, B: VT 50DPA versus FS 50DPA, C: VT 60DPA versus FS 60DPA, D: VT 80DPA versus FS 80DPA).

Gene ontology analysis results showed that the GO terms ‘polysaccharide biosynthetic process (GO:0000271)’, ‘pentose‐phosphate shunt’ (GO:0006098), ‘response to red light’ (GO:0010114), ‘response to blue light’ (GO:0009637), ‘cysteine biosynthetic process’ (GO:0019344) and ‘photosystem II assembly (GO:0010207)’ were more dominant under the biological processes. The main predominant functional molecular genes were the GO terms ‘chlorophyll binding’ (GO:0016168), ‘structural constituent of ribosome’ (GO:0003735) and ‘2 iron, 2 sulfur cluster binding’ (GO:0051537). The GO terms ‘plastoglobule’(GO:0010287), ‘photosystem I’ (GO:0009773),’plant‐type cell wall’ (GO:0009505), ‘chloroplast thylakoid membrane’ (GO:0009535) and anchored component of plasma membrane (GO:0046658) constitute the most common categories in the cellular component, in all the berry developmental stages(Fig. 5A–D).

**Fig. 6:**
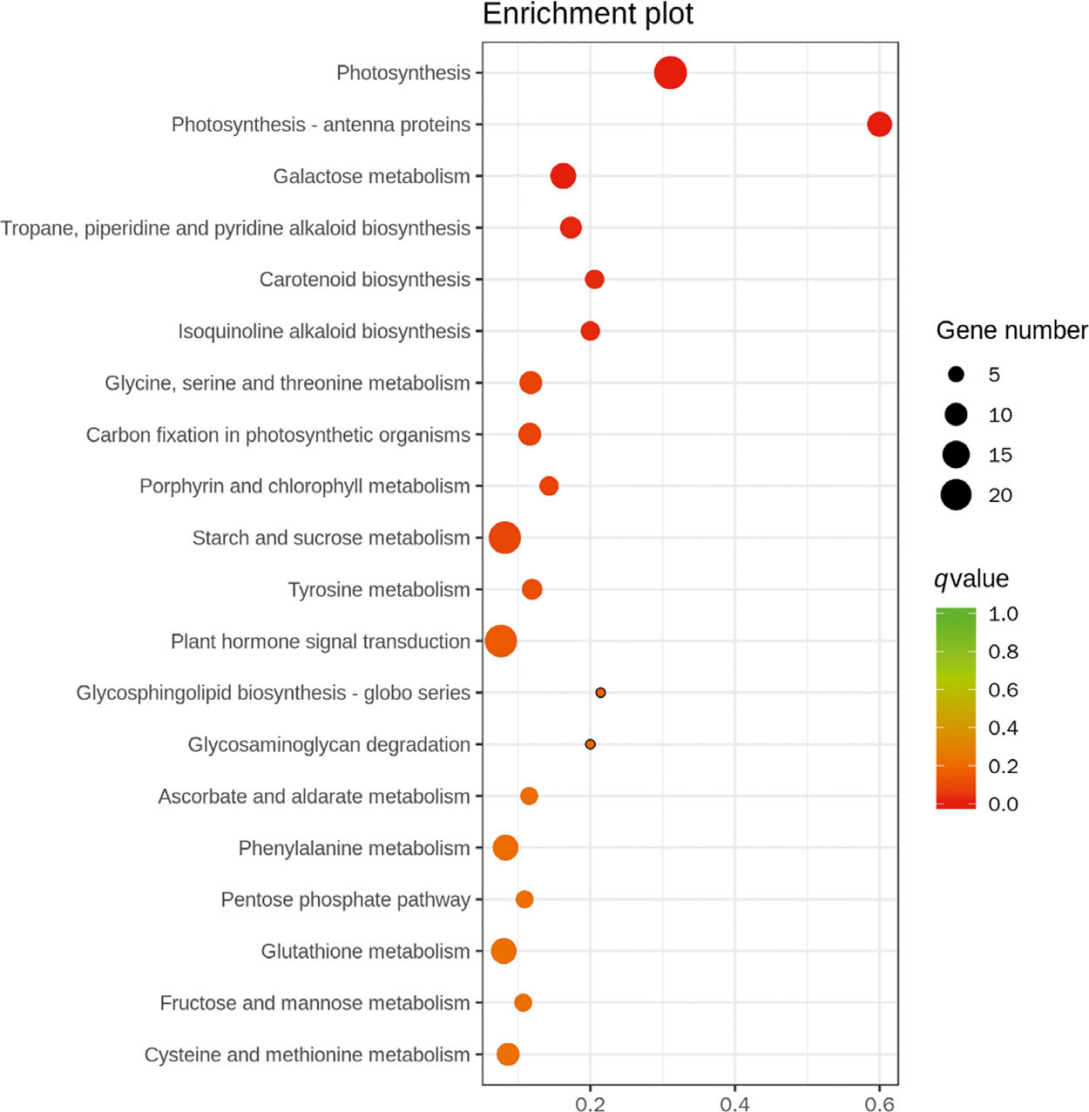
GOKEGG details of the most enriched groups.

In the KEGG classification, ‘Photosynthesis’ (ko00195), ‘Starch and sucrose metabolism’ (ko00500), ‘Galactose metabolism’ (ko00052) and ‘Photosynthesis‐antenna proteins’ (ko00196) were the most enriched groups (Fig. 6).

Several genes encoding for sugar accumulation, transport, and metabolism processes were identified in the two grape varieties. Notably, genes like SWEET2a (VIT_10s0003g02190), SWEET14 (VIT_17s0000g00820), SWEET10 (VIT_17s0000g00830), SWEET4 (VIT_02s0025g02080), and SWEET10 (VIT_17s0000g00830) were identified in the four grapefruit developmental phases. These genes are associated with sugar transport and accumulation. In addition, several transcription factors like thMYB44 were gradually up‐regulated as the grape berries developed. Equally, HXK related genes were identified, with them being highly expressed in FS cultivars than in the VT cultivar (Table S3).

### Real‐time RT‐PCR validation

**Fig. 7:**
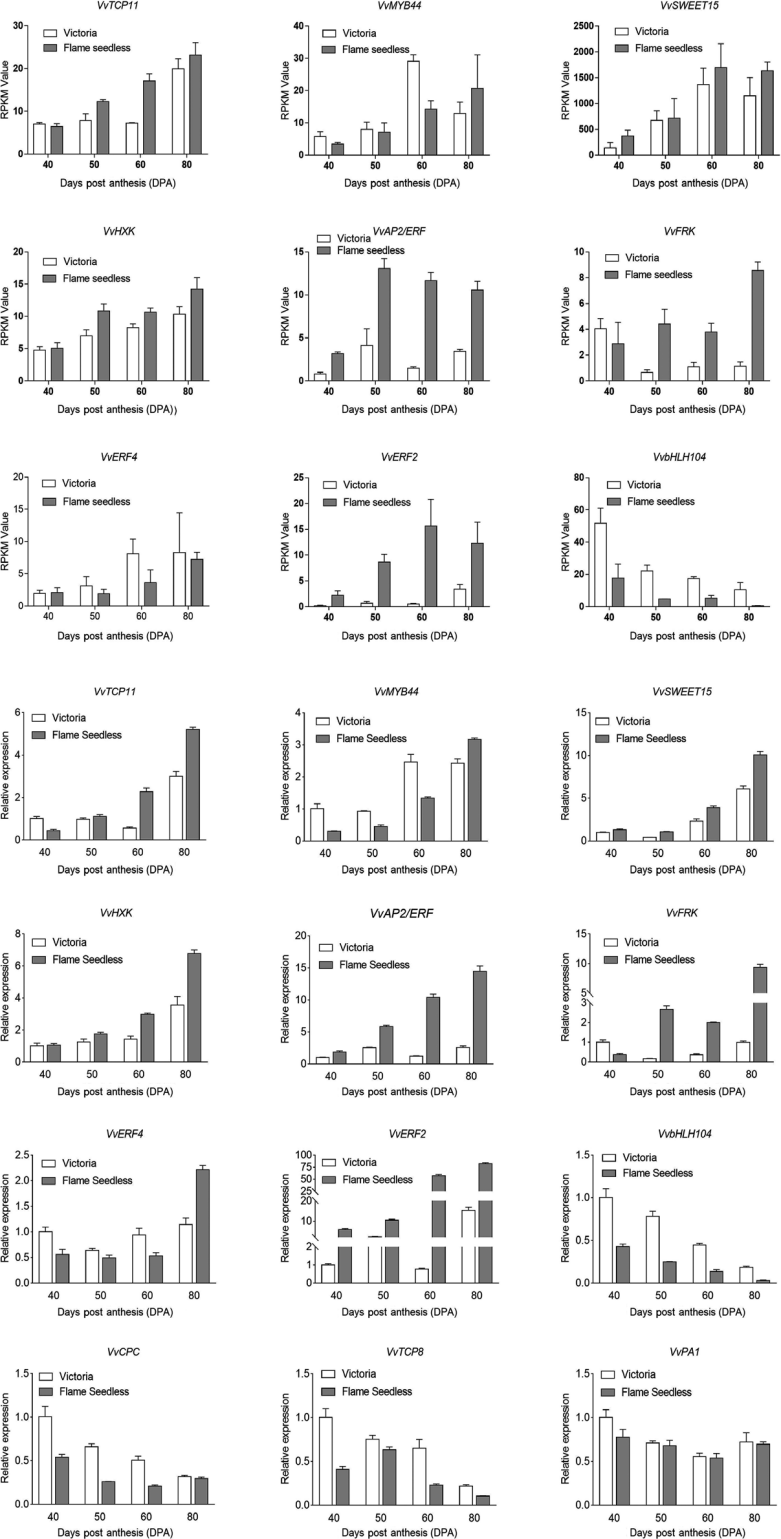
Real‐time PCR validation of DE transcripts. The white bar graphs represent the results for Victoria (VT), while gray bars indicate the results for Flame Seedless (FS). Bars represent mean ± SE (*n* = 3).

The qPCR was performed to evaluate the relative levels of twelve differently expressed genes in four developmental stages of VT and FS by RNA‐Seq using FPKM value method(a‐l) and by qPCR using the 2‐ΔΔCt method(m‐x). Gene‐specific primers were utilized. From qPCR analyses, there was an overall agreement of 90% indicating trends similarity of transcript abundances when evaluated by real‐time RT‐PCR (Fig. 7).

## Discussion

Grapevine (*Vitis vinifera* L.) is a nonclimacteric fleshy berry with wide economic utility. The developmental stages of these grape berries precede a double sigmoid curve with three main phases [34]. At the onset of berry ripening, the genes are expressed and metabolic activities associated with berry size, weight, and texture are subtly coordinated so as to achieve required organic acids, and subsequent accumulation of metabolites (both primary and secondary) like soluble sugars, aromas, and pigments[2]. At this initial developmental stage, Kennedy *et al*. [3] pointed out that the berry size increases up to nearly 70% of its total fruit volume. In this study, the berry size and diameter of VT and FS gradually increased as from 40 DAP up to 80 DAP, where it had attained its full growth size (Fig. 1). Notably though, the berry size of VT variety was considerably larger when compared to FS variety. This can be due to the previously described positive correlation between the grape berry weight and seeds in various grape cultivars [35, 36, 37, 38, 39], as it may be probably due to effects of growth regulators being produced by seeds [40, 41].

The second berry developmental phase majorly constitutes the veraison; a stage characterized by berry softening, accumulation of sugar, coloration, and renewed size increase. This phase denotes a very important phase in berries ripening because it indicates commencement of the fruit ripening processes. The accumulation of hexose sugars (i.e., glucose and fructose) is regarded as one of the main characteristic of berry development [42]. Among grapes, these soluble sugars content varies from species to species. For the 6‐year‐old cultivars of the Horticultural Crop Research Institute of Xinjiang Academy of Agricultural Sciences, the Flame Seedless grape variety’s soluble solids have the highest content, significantly compared to Victoria cultivars. The lowest content of Victoria grapevine was 61.8%. Hence, selected berries from the two cultivars were representative of berry development and ripening process and were suitable to be used in transcriptome analysis.

Overall, the major carbohydrate here is sucrose (Suc) that is transferred mainly from plant leaves to several plant tissues by Sugars Will Eventually be Exported Transporter (SWEET) efflux proteins and Suc transporters [43, 44]. Through this, sink cells absorb Suc or hexoses through their well‐equipped sugar transporters like disaccharide or monosaccharide transporters in roots, young leaves, seeds, and fruits [44, 45]. Several of these sucrose and monosaccharide transporter gene families have been characterized in *V. vinifera*. These sugar transporter families allow genes to be expressed specifically in various plant tissues and are also regulated during berry development [46]. For instance, the upregulation of SWEET sugar transporter genes in grapes has been shown to greatly contribute in glucose and fructose accumulation. Specifically, Zhang et al demonstrated that VvSWEET10, a grape SWEET gene, is strongly expressed during the grape’s veraison stage, and its upregulation contributed to an increase in the accumulation of soluble sugars in the berries [47]. From our study findings, VvSWEET genes were gradually up‐regulated during the postanthesis period, with the highest levels being observed at 60DPA and 80 DPA in both grape varieties (Fig. 7). Particularly, VvSWEET gene was highly up‐regulated in FS variety than in the VT varieties. Further, from our transcriptomic results, carbohydrate transport and metabolism were up‐regulated during berry development. In our results, we found 645 genes that were differentially expressed in all the four stages. In these genes, we identified 40 genes were related to carbohydrate transport and metabolism in eggNOG annotation (Table S4). In grapes, invertase catalyzes the conversion of Suc to its monosaccharide constituents Glc and Fru [48]. In Sultana berries, Hawker described that invertase enzyme increased immediately after flowering and that the activity peaked 6 to 7 weeks later, at véraison, when the rapid accumulation of hexoses commenced [49]. Herein, beta‐fructofuranosidase was identified among the differentially expressed genes present in the four developmental stages, and it may be associated with carbohydrate metabolism in VT and FS. Previously conducted study on *V. vinifera* showed that VvUFGT is the major gene that controls grape berry skin veraison [50, 51]. From our study findings, three genes (VIT_17s0000g07070, VIT_07s0151g00540, and VIT_17s0000g07030) were annotated as UDP‐glycosyltransferase, and their expression was up‐regulated during the stage of pulp veraison.

Further, hexokinases have been projected as being dual‐purpose enzymes in plants, with both catalytic and regulatory roles [52, 53, 54, 55, 56]. These plant HXKs phosphorylates fructose (Fruc), glucose (Glc), galactose, and mannose [57, 58]. Despite lack of a clear understanding of HXK roles as sugar sensor, it has been demonstrated that HXK‐dependent signaling functions can be achieved by HXK‐dependent sugar metabolism [59, 60]. From our findings, VvHXK was gradually expressed in both the VT and FS grape cultivars, over their developmental phases. Notably though, the VvHXK expression was highly expressed in FS cultivars, more than VT, all through the berry development (Fig. 7). In a previously reported study by Jang et al, overexpression of AtHXK genes in Arabidopsis thaliana led to hypersensitivity to sugars, while plants with antisense AtHXK RNA were sugar hyposensitive [59], hence implying that HXKs have a functional role in sugar‐sensing and/or signaling.

Also, from our findings, pectin catabolic processes were highly up‐regulated. Proportionally, nearly 35% of the primary cell wall of dicots is accounted by pectins, which are structurally complex polysaccharides [61]. Majorly, pectin is comprised of homogalacturonan (HG); a product that is de‐methylesterified by apoplastic pectin methyl esterases (PMEs) and been secreted into the cell wall [62, 63, 64]. The methylesterification HG is crucial for wall plasticity, tissue integrity, cell adhesion [65, 66, 67, 68, 69], and (both biotic and abiotic) stress responses [63, 70, 71]. PME activity is well regulated by endogenous pectin methylesterase inhibitors (PMEIs) belonging to the large multigene protein family PF04043 (http://pfam.xfam.org/family/PF04043) including invertase inhibitors (INHs). PMEI was first identified in kiwi fruits [72] and subsequently in several other fruits like tomato, banana, Arabidopsis, broccoli, pepper, and wheat [64, 73, 74, 75, 76, 77, 78, 79]. Recently, the role of PMEIs was demonstrated in a number of growth and development processes including apical meristems maturation [80], cell and tissue size [65, 81], growth acceleration [82], and fruit maturation and ripening [76, 78, 83]. Thus, in grape berries, pectins may be involved in maturation processes like shaping the berry size, ripening, and apical meristem growth of the grapevines.

Transcription factors (TFs) form a significant part of genes that regulates the transcription of their downstream target genes. These TFs have a DNA‐binding ability which is sequence specific that can be grouped into families based on their conserved motifs encoding their representative DNA‐binding domains [84]. In plants, some of the largest TFs are helix–loop–helix (HLH) family and TCP transcription factors. They play various significant roles. For instance, TCP transcription specificity factors have significant roles in various physiological and biological processes in plant growth and development. Thus far, the TCP gene family has been studied in various plants such as Arabidopsis [85], tomato [86], apple [87], strawberry [88], peach [89], and grapes [90]. Equally, several MYB transcription factors sub‐families have been identified in plants [91, 92], with R2R3‐MYB transcription factors being the most common. These MYB transcription factors have widely been associated with regulating plant growth, hormonal signal transduction, secondary metabolism, stress, and disease resistance [93, 94, 95]. Further, Wei *et al* demonstrated that FaMYB44.2, an MYB transcription factor, regulates organic acid content and soluble sugars in strawberry fruits [96]. Also, MaMYB3 transcription factor has been found to regulate fruit ripening in bananas [97]. In this study, the MYB44 was gradually up‐regulated as the fruit developed, but this transcription factor differed from the expression pattern reported by Lingzhi Wei [96], whereby there was a down‐regulated expression in the sugar metabolism pathway, which may be due to the different grape species utilized in these two studies. Further, bHLH14 transcription factor, which may be associated with glucose metabolism, was gradually down‐regulated as the berry developed. In a previously conducted study on bHLH gene family in Lotus (Nelumbo nucifera Gaertn.) under abiotic stress, Mao et al determined the expression levels of NnbHLH 104 as low in four different tissues [98]. In this study, VvbHLH104‐D was down‐regulated in VT and FS varieties during berry development (Table S5). Majority of these bHLH proteins characterized in Arabidopsis have been linked to several plant regulatory roles including their development, phytochrome signaling, fruit dehiscence, stress responses, and hormonal signaling [99].

In conclusion, our study findings contribute new knowledge to the available catalog of gene expression patterns for upcoming investigations whose aim is to dissect the transcriptional regulatory hierarchies of developmental stages of berries in a widely growing grape cultivars as wee as in other nonclimacteric fruits.

## Conflict of interest

The authors declare no conflict of interest.

## Author contribution

Conceptualization, HZ and FZ; Data curation, HZ, FZ, MP, and XW; Formal analysis, HZ, FZ, MP, WZ, HX, XZ; Funding acquisition, MP; Methodology, HZ, FZ, and XZ; Project administration, HZ and MP; Resources, FZ, SH, CA and MW; Supervision, MP and TH; Validation, HZ, FZ and MW; Writing – original draft, HZ; Writing – review & editing, HZ, MP and TH.

## Supporting information


**Table S1.** qPCR Primers.
**Table S2.** Statistical table summary of all sequenced data for the various berry developmental stages.
**Table S3.** Statistical table of sequence comparison results of sample sequencing data and selected reference genome.

**Table S4.** A list of 40 common carbohydrate genes in VT and FS, at maturity.

**Data S1.** Gene Annotation data of 40DPA, 50DPA, 60DPA and 80DPA grape berry developmental stages.

## Data Availability

Data can be availed by the corresponding author, upon reasonable request.
