# Supplementary material for: Comparative phenotypic and transcriptomic analysis of Victoria and flame seedless grape cultivars during berry ripening
Source: FEBS Open Bio. 2020 Nov 4;10(12):2616–30. doi: 10.1002/2211-5463.12996 (PMC7714085; doi:10.1002/2211-5463.12996)
Supplement: Supplementary file 1 — Table S1. qPCR Primers. Table S2. Statistical table summary of all sequenced data for the various berry developmental stages. Table S3. Statistical table of sequence comparison results of sample sequencing data and selected reference genome. [file FEB4-10-2616-s001.pdf]

Supplementary Table 1: List of primers used in this study.

Gene	Primer Name	Primer Sequence
vvERF	VvERF4-F1.1	TCCAGAAAAGCCGAAGACCC
	VvERF4-R1.2	ATCGAGATTGAGCAGCCTGG
	VvERF4-F2.1	GCCAATTTTCCGCAGTTCGT
	VvERF4-R2.2	CAGGGTGAAAATCGGCAACC
VvMYB44	VvMYB44-F1.1	CCGATGACCTTTGGTTCGGA
	VvMYB44-R1.2	CTCAATCGCCCATGCCAATG
VvMYB44	VvMYB44-F2.1	CTCCCTCCCTGGAACTGAGA
	VvMYB44-R2.2	TCCGAACCAAAGGTCATCGG
VvRAP2	VvRAP2-11-F1.1	CAAGTTTGTTGGGGTGAGGC
	VvRAP2-11-R1.2	TGGAACCACGAAGAAGGCAG
VvRAP2	VvRAP2-11-F2.1	ACCCTGCAAACAGACCAAGG
	VvRAP2-11-R2.2	AGTGTTGAATGTCCCCAGCC
VvERF5	VvERF5-F1.1	GGCGCTACAGTCTTGGATGA
	VvERF5-R1.2	TGTTCCACGTGTGGTTTCCA
VvERF5	VvERF5-F2.1	CATCGCGATTCCTCACCCTT
	VvERF5-R2.2	GTGCCAAGCCAAACCCTAGA
VvERF045	VvERF045-F1.1	CCCTTACAATCCCAACGCCT
	VvERF045-R1.2	TTGTGGCGACATGGGAAGTC
VvERF045	VvERF045-F2.1	GAGGGCGAGAACCAACTTCC
	VvERF045-R2.2	GTGGCTTTTGAGGCTCATGC
VvSWEET15	VvSWEET15-F1.1	GAGAAGAAGCTGCCGGAACA
	VvSWEET15-R1.2	TCTCGCGATTCCCAGTTTCT
VvSWEET15	VvSWEET15-F2.1	GGGTCATATTGGGGCTGGTC
	VvSWEET15-R2.2	CCATTCTGCTGGGCTCCGAT
VvETC1	VvETC1 -F1.1	AGGTGGGCTCTAATTGCTGG
	VvETC1 -R1.2	CCAACTCTCCAATAAACCCTTGC
VvETC1	VvETC1 -F2.1	GTGGGCTCTAATTGCTGGGA
	VvETC1 -R2.2	CCTCCAACTCTCCAATAAACCCT
VvbHLH104	VvbHLH104-F1.1	TCATGCTTCCACTCCACTAGC
	VvbHLH104-R1.2	AAGAAGATCCCAACCACCGTC
VvbHLH104	VvbHLH104-F2.1	AGAGAGGCGTCACTGCAAAT
	VvbHLH104-R2.2	TGGGTTGTTTTGTGCTAGTGG
Vv bHLH137	Vv bHLH137-F1.1	AAGGGGCAGCAAAATCAATGG
	VvbHLH137-R1.2	TGATCCACCACAGGCTCTTG
Vv bHLH137	VvbHLH137-F2.1	GGGCAGCAAAATCAATGGAGG

	Vv bHLH137-R2.2	GGTTGATCCACCACAGGCTC
VvFKL-2	VvFKL-2-F1.1	TTGTTGGGAAGCTCGGAGAC
	VvFKL-2-R1.2	ATGAACTCACGCTCTCCGTC
VvFKL-2	VvFKL-2-F2.1	GAGTTTCGGCGAGATGCTGA
	VvFKL-2-R2.2	AGCTTCCCAACAAACGCAGA
VvTCP11	VvTCP11-F1.1	GCAACCGCTTACGTCAGTTC
	VvTCP11-R1.2	CCCAGACTGCAACAACAACG
VvTCP11	VvTCP11-F2.1	TACAAAGGTGAACGGCCGAG
	VvTCP11-R2.2	CGGTAGCGGCTATGATGGAG
VvERF2	VvERF2 -F1.1	CGTCGAGAGGGAGGCATTAC
	VvERF2 -R1.2	CCGATAAGCTGCTCGGTCAT
VvERF2	VvERF2 -F2.1	AGTTCGCTGCGGAGATTAGG
	VvERF2 -R2.2	CCCTGTGCGGAAAATTGAGC
VvHEX3	VvHEX3-F1.1	GTGTGATGTAGTGACCCGCA
	VvHEX3-R1.2	CCATCCATTGCTACCACCGT
VvHEX3	VvHEX3-F2.1	AAAGGTGAGAAAGCTCGTCGT
	VvHEX3-R2.2	CCATCACTTCCACGACTCCT
Vv TCP8	Vv TCP8-F1.1	TATGCCAGCTATCTGTGCCG
	Vv TCP8-R1.2	CGAAAAGTTCGCCGGAATCG
Vv TCP8	VvTCP8-F2.1	TTCCGGCGAACTTTTCGACT
	Vv TCP8-R2.2	CCAAGATTCTCCGGCTTGGA
VvPur	VvPur-F1.1	ACGTGGAGCTGCTGTGTAAA
	VvPur-R1.2	AAGAACCAGGAAATGCCGCT
VvPur	VvPur-F2.1	ATCTGGTGCCCCTCAACTTC
	VvPur-R2.2	ACGTCATTTCCGCCTGAGTT
VvERF1B	VvERF1B -F1.1	GCTCCACTCAGGAAACCTCA
	VvERF1B-R1.2	ATGCCTGGTCGAGTCCCTTA
VvERF1B	VvERF1B-F2.1	CTCGACCAGGCATGGCATAA
	VvERF1B-R2.2	GCACTTGATCTCCTGCATCG
VvWRKY31	VvWRKY31 -F1.1	AACCCAACTCAGAACCTCGC
	VvWRKY31 -R1.2	TGGTGGTGGGTCGGTAATTG
VvWRKY31	VvWRKY31 -F2.1	CCCAACCCAACTCAGAACCT
	VvWRKY31 -R2.2	TGGTGGGTCGGTAATTGAGC
VvERF027	VvERF027-F1.1	ACTCGAATATGGCTCGGCAC
	VvERF027-R1.2	CGTATATCGCTTGCGGAGGT
VvERF027	VvERF027-F2.1	AAAAGGCACTGATGCCACCT
	VvERF027-R2.2	GTGTTTGGGCTCTCCGAACT
VvERF003	VvERF003-F1.1	TGCTACATGGCATCACCTCA
	VvERF003-R1.2	CCTGTACTTGCCGTCGTCTC

VvERF003	VvERF003-F2.1	TACATGGCATCACCTCAAGGG
	VvERF003-R2.2	CTGTACTTGCCGTCGTCTCT
VvWRKY48	VvWRKY48-F1.1	GTGGAGCGATCCTCCGATG
	VvWRKY48-R1.2	CATGGCGAATGAGGAAGCAG
VvWRKY48	VvWRKY48-F2.1	AGCTACTATCGTTGCACCACT
	VvWRKY48-R2.2	CGGCGGAATCCCAAGACTTC
VvATP6	VvATP6-F1.1	AGCGCATCAATGAGAGTCGG
	VvATP6-R1.2	TATCCCCATCCAGCAACCCA
VvATP6	VvATP6-F2.1	TAACCGACAGGATGTGGGCA
	VvATP6-R2.2	GGTCAACTGGCTTGTGGTCA
VvSS2	VvSS2 -F1.1	ATCTGGTTGTGGTAGCTGGT
	VvSS2 -R1.2	GTTCACCATTACGTGCTCGG
VvSS2	VvSS2 -F2.1	GGCAAATCTGGTTGTGGTAGC
	VvSS2 -R2.2	TACGTGCTCGGTTTGTTTGG
VvERF011	VvERF011-F1.1	GCCTCAACTTCCCGGAGTAT
	VvERF011-R1.2	GGTTTGTCGGAGACTCTGCT
VvERF011	VvERF011-F2.1	GGTCCAGGATATGGCTTGGT
	VvERF011-R2.2	ATGGATGCGGCGGATAAGTC
VvWRKY32	VvWRKY32-F1.1	AGCTGGGGTGTGTCATCAAG
	VvWRKY32-R1.2	TGGACTTGGAGCCGATTGAA
VvWRKY32	VvWRKY32-F2.1	AAGCTGGGGTGTGTCATCAA
	VvWRKY32-R2.2	GTTGGACTTGGAGCCGATTG
VvUDP2	VvUDP2 -F1.1	TGACCATTTGGGATGGGGTG
	VvUDP2 -R1.2	TTTGGAAGTGTTGCGGCTTG
VvUDP2	VvUDP2 -F2.1	ACCATTTGGGATGGGGTGTC
	VvUDP2 -R2.2	CCTTTGGAAGTGTTGCGGC
VvGAPDH	VvGAPDH-F	TTCTCGTTGAGGGCTATTCC
	VvGAPDH-R	CCACAGACTTCATCGGTGACA

Supplementary Table 2: Statistical table summary of all sequenced data for the various berry developmental stages.

Samples	Clean reads	Clean bases	GC Content	%$^{\geqq Q30}$
VT 40^{-a}	27,528,195	8,235,092,158	45.88%	94.31%
VT 40^{-b}	29,810,916	8,869,186,196	46.18%	92.66%
VT 40^{-c}	27,568,221	8,223,425,062	46.37%	92.51%
FS 40^{-a}	94,239,829	28,183,441,978	47.09%	89.44%
FS 40^{-b}	28,997,172	8,669,964,788	47.13%	89.55%
FS 40^{-c}	24,465,455	7,315,402,268	47.15%	89.47%
VT 50^{-a}	40,891,236	12,234,674,718	47.00%	88.74%
VT 50^{-b}	22,645,557	6,779,218,254	47.05%	88.98%
VT 50^{-c}	31,820,780	9,443,626,630	47.04%	93.75%
FS 50^{-a}	24,971,324	7,469,279,886	47.27%	91.31%
FS 50^{-b}	25,552,192	7,643,351,398	47.09%	90.54%
FS 50^{-c}	24,263,718	7,261,002,804	47.35%	91.20%
VT 60^{-a}	27,390,408	8,190,348,158	47.10%	89.34%
VT 60^{-b}	24,377,899	8,186,362,356	47.49%	90.88%
VT 60^{-c}	22,688,622	6,757,265,852	47.97%	92.96%
FS 60-a	24,576,690	7,355,413,916	47.15%	91.31%
FS 60-b	22,577,185	6,753,758,482	46.92%	90.40%
FS 60-c	32,937,677	9,859,594,892	46.73%	90.43%
VT 80^{-a}	22,589,176	6,758,961,572	47.31%	94.85%
VT 80^{-b}	27,497,768	8,228,517,094	47.24%	94.83%
VT 80^{-c}	29,377,965	8,794,339,582	47.62%	94.75%
FS 80-a	29,406,732	8,790,221,222	47.42%	89.12%
FS 80-b	23,615,492	7,066,691,604	46.97%	89.54%
FS 80-c	30,154,330	9,023,335,588	46.87%	88.99%

Supplementary Table 3: Statistical table of sequence comparison results of sample sequencing data and selected reference genome

Samples	Total Reads	Mapped Reads	Uniq Mapped Reads	Mutiple Map Reads	Reads Map to '+'	Reads Map to'-'
VT 40[–a]	55,056,390	34,469,353 (62.62%)	34,032,797 (61.81%)	436,556 (0.79%)	17,200,649 (31.24%)	17,179,336 (31.20%)
VT 40[–b]	59,621,832	36,923,353 (61.93%)	36,445,748 (61.13%)	477,446 (0.80%)	18,417,109 (30.89%)	18,405,468 (30.87%)
VT 40[–c]	55,136,442	31,910,439 (57.88%)	31,514,407 (57.16%)	396,032 (0.72%)	15,921,784 (28.88%)	15,906,975 (28.85%)
FS 40[–a]	188,479,658	148,880,948 (78.99%)	146,626,544 (77.79%)	2,254,404 (1.20%)	67,084,338 (35.59%)	66,785,445 (35.43%)
FS 40[–b]	57,994,344	46,506,635 (80.19%)	45,895,261 (79.14%)	612,374 (1.06%)	23,240,816 (40.07%)	23,147,446 (39.91%)
FS 40[–c]	48,930,910	38,926,333 (79.55%)	38,417,470 (78.51%)	508,863 (1.04%)	19,456,236 (39.76%)	19,372,849 (39.59%)
VT 50[–a]	81,782,472	60,889,227 (74.45%)	60,136,610 (73.53%)	752,617 (0.92%)	30,443,944 (37.23%)	30,310,144 (37.06%)
VT 50[–b]	45,291,114	34,597,551 (76.39%)	34,192,321 (75.49%)	405,230 (0.89%)	17,282,789 (38.16%)	17,235,850 (38.06%)
VT 50[–c]	63,641,560	44,375,058 (69.73%)	43,851,552 (68.90%)	523,506 (0.82%)	22,138,035 (34.79%)	22,128,163 (34.77%)
FS 50[–a]	49,942,648	40,321,787 (80.74%)	39,788,268 (79.67%)	533,519 (1.07%)	20,139,419 (40.33%)	20,087,561 (40.22%)
FS 50[–b]	51,104,384	41,457,681 (81.12%)	40,928,923 (80.09%)	528,758 (1.03%)	20,696,396 (40.50%)	20,665,457 (40.44%)
FS 50[–c]	48,527,436	39,586,761 (81.58%)	39,050,161 (80.47%)	536,600 (1.11%)	19,765,468 (40.73%)	19,729,685 (40.66%)
VT 60[–a]	54,780,816	40,950,576 (74.75%)	40,441,646 (73.82%)	508,930 (0.93%)	20,435,962 (37.30%)	20,406,756 (37.25%)
VT 60[–b]	54,755,798	40,631,411 (74.20%)	40,146,528 (73.32%)	484,883 (0.89%)	20,277,388 (37.03%)	20,250,629 (36.98%)

VT 60^{-c}	45,377,244	33,704,433 (74.28%)	33,305,105 (73.40%)	399,328 (0.88%)	16,812,013 (37.05%)	16,806,394 (37.04%)
FS 60-a	49,153,380	40,248,944 (81.88%)	39,709,158 (80.79%)	539,786 (1.10%)	20,082,970 (40.86%)	20,075,913 (40.84%)
FS 60-b	45,154,370	37,435,919 (82.91%)	36,961,361 (81.86%)	474,558 (1.05%)	18,690,383 (41.39%)	18,661,575 (41.33%)
FS 60-c	65,875,354	54,521,034 (82.76%)	53,771,544 (81.63%)	749,490 (1.14%)	27,234,537 (41.34%)	27,159,627 (41.23%)
VT 80^{-a}	45,178,352	37,507,392 (83.02%)	36,981,444 (81.86%)	525,948 (1.16%)	18,696,014 (41.38%)	18,729,317 (41.46%)
VT 80^{-b}	54,995,536	38,730,272 (70.42%)	38,118,587 (69.31%)	611,685 (1.11%)	19,312,148 (35.12%)	19,314,782 (35.12%)
VT 80^{-c}	58,755,930	43,790,445 (74.53%)	43,134,615 (73.41%)	655,830 (1.12%)	21,831,811 (37.16%)	21,845,417 (37.18%)
FS 80-a	58,813,464	46,773,822 (79.53%)	46,155,807 (78.48%)	618,015 (1.05%)	23,56,985 (39.71%)	23,326,152 (39.66%)
FS 80-b	47,230,984	38,043,311 (80.55%)	37,535,737 (79.47%)	507,574 (1.07%)	18,980,649 (40.19%)	18,984,122 (40.19%)
FS 80-c	60,308,660	48,751,362 (80.84%)	48,116,470 (79.78%)	634,892 (1.05%)	24,331,052 (40.34%)	24,327,578 (40.34%)
